# Partial Support Ventilation and Mitochondrial-Targeted Antioxidants Protect against Ventilator-Induced Decreases in Diaphragm Muscle Protein Synthesis

**DOI:** 10.1371/journal.pone.0137693

**Published:** 2015-09-11

**Authors:** Matthew B. Hudson, Ashley J. Smuder, W. Bradley Nelson, Michael P. Wiggs, Kevin L. Shimkus, James D. Fluckey, Hazel H. Szeto, Scott K. Powers

**Affiliations:** 1 Department of Kinesiology, Temple University, Philadelphia, Pennsylvania, United States of America; 2 Department of Applied Physiology and Kinesiology, Center for Exercise Science, University of Florida, Gainesville, Florida, United States of America; 3 Department of Natural Sciences, Ohio Dominican University, Columbus, Ohio, United States of America; 4 Department of Health and Kinesiology, Texas A&M University, College Station, Texas, United States of America; 5 Department of Pharmacology, Weill Cornell Medical College, New York, New York, United States of America; University of Louisville School of Medicine, UNITED STATES

## Abstract

Mechanical ventilation (MV) is a life-saving intervention in patients in respiratory failure. Unfortunately, prolonged MV results in the rapid development of diaphragm atrophy and weakness. MV-induced diaphragmatic weakness is significant because inspiratory muscle dysfunction is a risk factor for problematic weaning from MV. Therefore, developing a clinical intervention to prevent MV-induced diaphragm atrophy is important. In this regard, MV-induced diaphragmatic atrophy occurs due to both increased proteolysis and decreased protein synthesis. While efforts to impede MV-induced increased proteolysis in the diaphragm are well-documented, only one study has investigated methods of preserving diaphragmatic protein synthesis during prolonged MV. Therefore, we evaluated the efficacy of two therapeutic interventions that, conceptually, have the potential to sustain protein synthesis in the rat diaphragm during prolonged MV. Specifically, these experiments were designed to: 1) determine if partial-support MV will protect against the decrease in diaphragmatic protein synthesis that occurs during prolonged full-support MV; and 2) establish if treatment with a mitochondrial-targeted antioxidant will maintain diaphragm protein synthesis during full-support MV. Compared to spontaneously breathing animals, full support MV resulted in a significant decline in diaphragmatic protein synthesis during 12 hours of MV. In contrast, diaphragm protein synthesis rates were maintained during partial support MV at levels comparable to spontaneous breathing animals. Further, treatment of animals with a mitochondrial-targeted antioxidant prevented oxidative stress during full support MV and maintained diaphragm protein synthesis at the level of spontaneous breathing animals. We conclude that treatment with mitochondrial-targeted antioxidants or the use of partial-support MV are potential strategies to preserve diaphragm protein synthesis during prolonged MV.

## Introduction

Mechanical ventilation (MV) is used to provide adequate pulmonary ventilation in patients that require ventilatory support arising from respiratory failure, drug overdose, spinal cord injury, and surgery. Two different modes of MV are commonly used clinically: 1) full-support MV; and 2) partial-support MV. During full-support MV the ventilator performs all of the work of breathing and the patient’s inspiratory muscles are inactive. In contrast, during partial support MV the ventilator provides a portion of the work of breathing and the patient’s inspiratory muscles provide the remainder. Unfortunately, full-support MV results in the rapid development of diaphragm weakness due to both myofiber atrophy and contractile dysfunction, a condition commonly referred to as ventilator-induced diaphragm dysfunction (VIDD) [1]. The development of VIDD is important because diaphragmatic weakness is predicted to contribute to problems in weaning patients from MV, and therefore developing clinical interventions to prevent MV-induced diaphragm weakness is essential [2–4]. Additionally, one of the most severe consequences of ventilating critically ill patients is impaired respiratory muscle function [5–7]. In fact, evidence in ventilated critically ill patients demonstrates that severe diaphragm weakness is associated with poor patient outcomes, further demonstrating the need for clinical interventions [4].

Similar to other forms of disuse muscle atrophy, VIDD occurs because of an imbalance between the rates of protein degradation and protein synthesis [8–13]. Previously, our group and others have demonstrated that as few as 12 hours of full-support mechanical ventilation results in increased proteolysis in the diaphragm [10, 14–21]. Further, prolonged full-support MV promotes a rapid decrease in protein synthesis in the diaphragm beginning as early as 6 hours after the initiation of MV [8, 22]. Therefore, the ideal strategy to prevent MV-induced diaphragmatic atrophy would be to prevent both the MV-induced decrease in protein synthesis and increase in proteolysis in the diaphragm.

Numerous studies have explored therapeutic strategies to impede MV-induced protease activation in the diaphragm and several pharmacological approaches can delay the rate of MV-induced diaphragm proteolysis [19, 23–25]. For example, we previously demonstrated that full support MV results in increased mitochondrial ROS production in the diaphragm and treatment with the mitochondrial-targeted antioxidant SS-31 can prevent both the MV-induced increase in mitochondrial ROS emission and the increase in proteolysis in the diaphragm [19, 21]. Further, this prevention of mitochondrial generated ROS prevented diaphragm muscle atrophy and contractile dysfunction. However, to date, no studies have investigated the effects of mitochondrial antioxidants on maintaining diaphragm muscle protein synthesis during full support MV.

Also, in addition to pharmacological approaches, another therapeutic approach that has the potential to delay the rate of MV-induced proteolysis is the use of partial-support MV which permits diaphragm contractile activity, albeit at lower activation levels than required for spontaneous breathing. Previously, we reported that compared to full support MV, partial-support MV delays the activation of proteolytic systems in the diaphragm as well as diaphragm atrophy and contractile dysfunction [26]. Further, a previous report investigating the effects of partial-support MV on maintaining diaphragm protein synthesis showed positive results [22]. Unfortunately, currently during prolonged MV there is no established therapeutic intervention to maintain protein synthesis in the diaphragm. Thus, developing therapeutic strategies, including mitochondrial antioxidant treatment or partial-support MV to prevent the MV-induced decrease in protein synthesis is important and forms the rationale for the current experiments.

Using an animal (rat) model of MV that exhibits diaphragm dysfunction similar to that observed in the human diaphragm during full-support MV, these experiments investigated two independent strategies that have the potential to sustain anabolic signaling and maintain protein synthesis in the diaphragm during prolonged MV. First, we replaced full-support MV with partial support MV to test the hypothesis that even low levels of diaphragm contractile activity can maintain diaphragm protein synthesis during prolonged MV. We also tested a second hypothesis that treatment of animals with a mitochondrial-targeted antioxidant will prevent MV-induced diaphragmatic oxidative stress and protect the diaphragm against MV-induced decreases in protein synthesis. This hypothesis was formulated from the knowledge that oxidative stress can blunt anabolic signaling and decreases protein synthesis in cells [27–29].

## Methods

### Experimental Animals

Adult female Sprague-Dawley rats were used in these experiments. Animals were maintained on a 12:12 hour light-dark cycle and provided food and water *ad libitum* throughout the experimental period. The Institutional Animal Care and Use Committee of the University of Florida approved these experiments.

### Experimental Design

These experiments determined the effectiveness of two therapeutic interventions to protect against full-support MV-induced decreases in diaphragm muscle protein synthesis. Specifically, these experiments were designed to achieve two objectives: 1) To determine if partial-support MV (when a patient’s inspiratory muscles provide a portion of the work of breathing) can maintain diaphragm protein synthesis at levels comparable to spontaneous breathing; and 2) To establish if treatment of animals with a mitochondrial-targeted antioxidant can prevent MV-induced oxidative stress and protect against the decreased diaphragmatic protein synthesis that occurs during prolonged full-support MV. To achieve these objectives adult female Sprague-Dawley rats were randomly assigned to one of the following groups: 1) Anesthetized and spontaneously breathing (SB); 2) Full-support MV using controlled mechanical ventilation (CMV); 3) Partial support MV using a pressure support mode (PSV) of MV; and 4) Exposed to CMV and treated with the mitochondrial-targeted antioxidant SS-31 to prevent MV-induced oxidative stress (CMVSS). All groups were exposed to a 12-hour treatment period (N = 8/group). At the completion of the experimental periods the animals were immediately sacrificed and the costal diaphragm quickly removed and stored at -80°C for subsequent analyses.

### Spontaneous Breathing

The SB animals breathed spontaneously during the duration of the experiment and served as time-matched controls for the MV groups. The SB animals were not mechanically ventilated, but received the same anesthetic dose, surgical intervention, and technical care as the MV animals.

### Mechanical ventilation

All animals assigned to full-support MV (CMV) and partial support ventilation (PSV) were acutely anesthetized with sodium pentobarbital (60 mg/kg (intraperitoneal) (IP)). Specific experimental details of our animal model of CMV has been described in detail previously (25).

### Partial support mechanical ventilation

Animals on PSV were acutely anesthetized with sodium pentobarbital (60 mg/kg IP). After reaching a surgical plane of anesthesia, the animals were tracheostomized and ventilated using partial-support ventilation as previously described [26].

### Mitochondrial-targeted antioxidant administration

Animals assigned to the CMVSS group were anesthetized and exposed to CMV using the aforementioned procedures. We selected the mitochondria-targeted antioxidant designated as “SS-31” for use in these experiments. This molecule belongs to a family of small, water-soluble peptides that contain an alternating aromatic-cationic motif (D-Arg-2’6’dimethylTyr-Lys-Phe-NH_2_) that has been shown to selectively target and concentrate ~5000-fold on the inner mitochondrial membrane [30, 31]. Furthermore, numerous studies have shown that SS-31 can selectively scavenge mitochondrial ROS and protect mitochondrial function [32]. The mitochondrial-targeted antioxidant SS-31 was administered as previously described [19]. Briefly, SS-31 was dissolved in saline and delivered in a bolus (loading) dose (3 mg/kg, subcutaneous injection) and a constant intravenous infusion (0.05 mg/kg/hr) of SS-31 was maintained throughout MV.

### Assessment of the work of breathing during partial-support MV

To determine the level of breathing support provided by the ventilator during PSV, we measured the inspiratory muscle time-tension index in both spontaneous breathing rats and animals undergoing PSV as previously described [26]. Briefly, we measured the inspiratory pressure generated and the temporal components of the breathing cycle in four rats that were switched back and forth between spontaneous breathing and PSV.

This was performed by obtaining pressure measurements while the animal was connected to ventilator and receiving PSV and measuring peak negative inspiratory pressure prior to ventilator triggering. After these measures were obtained, the animal was disconnected from the ventilator and peak negative inspiratory pressures were measured during spontaneous breathing. This was achieved using a technique described by Mortola and colleagues [33] in which the animal was connected to a non-rebreathing valve (Hans Rudolf model #2300 valve, Shawnee, KS). Specifically, the upper airway pressures generated during inspiration were measured using a rapid response and sensitive pressure transducer (AutoTran, Eden Prairie, MN). Using mean values for 30–50 breathing cycles the inspiratory muscle time-tension index was computed as (Ti/Ttot)x(Pneg/Pimax) where Ti is the inspiratory time, Ttot is the total duration of the respiratory cycle, Pneg is inspiratory pressure and Pmax is the estimated maximal inspiratory pressure generated by the respiratory system in rats (i.e., −80 cmH_2_O) [34].

### *In Vivo* measurement of protein synthesis

For the determination of rate of mixed muscle protein synthesis, [ring-^2^H_5_]- phenylalanine (priming dose, 3.945 μmol ∙ kg−1 followed by a continuous infusion rate, 0.1013 μmol ∙ kg−1 min−1, Cambridge Isotope Laboratories, Andover, MA) was administered intravenously during the last 6 hours of each experimental period using previously described techniques from our group [8]. For the isolation of protein-bound amino acids, 20 mg of muscle was homogenized and mixed muscle proteins precipitated by 10% trichloroacetic acid (TCA) and purified by centrifugation at 1000 x g. Precipitation and centrifugation was repeated for a total of 3 times. Samples were washed with water followed by the addition of ethanol which was evaporated until dry at 100°C. The resulting protein bound amino acids were hydrolyzed in 400 μL of 6 N HCL and heated at 100°C for 24 hours. Arterial samples at 0.5, 2, 4, 6 h throughout the protocol were measured to verify plateau enrichment of the precursor pool. Plasma samples were de-proteinized with 1:1 ratio of 15% sulfosalicylic acid, and amino acids separated by centrifugation at 10,000 x g. Before derivatization, all samples were washed over a cation exchange column (Dowex AG 50W-8X, 100–200 mesh, H+ form, Sigma-Aldrich, St. Louis, MO) eluted from the column with 2 N ammonium hydroxide, collected and dried under a vacuum (Savant SpeedVac, ThermoFisher Scientific, Waltham, MA). Once dried, samples were derivatized at 70°C for 4 hours with 100 μL of acetonitrile and N-methyl-N-(t-butyldimethylsilyl) trifluoroacetamide (MTBSTFA, Pierce Chemical, Rockford, IL) at a 1:1 ratio.

The tracer:tracee ratio for [ring-^2^H_5_]-phenylalanine:phenylalanine was determined by gas chromatography/mass spectrometry (model GC-7820 coupled with MS-5975, Agilent Technologies, Wilmington, DE) under electron impact and selective ion recording [35]. For phenylalanine, m/z 234 (m + 0), 237 (m +3), and 239 (m + 5) were monitored, with m+0 representing the lowest molecular weight of the ion and m+5 representing [ring-^2^H_5_]-phenylalanine. Plasma enrichment is determined by the ratio of m+5/m+0 and protein bound enrichment will be determined by m+5/m+3 in order to increase sensitivity due to relatively low abundance of m+5 [36]. The fractional rate of protein synthesis (FSR) per hour was calculated as the incorporation of stable isotope into the protein bound pool of muscle divided by the plasma concentration at the final time point as a function of time: FSR = E_t_ ∙ (E_p_ x t) * 100 where t is the time, in hours, the tissue was subjected to tracer, and E_t_ and E_p_ are the enrichments of [ring-^2^H_5_]-phenylalanine in hydrolyzed tissue protein an in plasma amino acids, respectively.

### Western Blot Analysis

Protein content was compared across groups via Western Blot analysis using previously described methods [12, 16]. 4-hydroxynonenal (4-HNE) conjugated proteins were measured as an index of oxidative damage (Abcam, Cambridge, MA; product #46546). To measure markers of protein synthesis signaling, membranes were incubated with antibodies against phosphorylated Akt (Ser 473), phosphorylated proline rich Akt substrate of 40kD (PRAS40) (Thr 246), phosphorylated mammalian target of rapamycin (mTOR) (Ser 2448), and phosphorylated 4E-binding protein 1 (4E-BP1) (Thr 37/46), all purchased from Cell Signaling Technology (Danvers, MA). Subsequently, membranes were stripped, blocked, and incubated with antibodies against total Akt, GSK3β, PRAS40, mTOR, ribosomal protein s6, and 4E-BP1 all purchased from Cell Signaling Technologies (Danvers, MA). For all western blots where both phosphorylated protein and total protein were measured, the data were analyzed as a ratio of the phosphorylated to total protein ratio by dividing the density of phosphorylated protein by the density of total protein. All western blots were normalized to α-tubulin as a loading control. Product numbers for specific antibodies from Cell Signaling Technologies are as follows: Akt (#2920), pAkt (#4058), PRAS40 (#2691), PRAS40 (#2997), p-mTOR (#5536), mTOR (#2983), 4E-BP1 (#9452), and p-4E-BP1 (#9459).

### Statistical Analysis

Group sample size was determined via use of a power analysis of preliminary data from our laboratory. Comparisons between groups were made by a one-way ANOVA, and when appropriate a Student-Newman-Keuls test was performed post hoc. Note that due to the large number of groups in the experimental design, western blot data were analyzed only within the same time period. Significance was established at p < 0.05.

## Results

### Diaphragm activation during PSV

Prior to beginning these experiments, we established the level of ventilatory support provided to the experimental animals during PSV. Specifically, to determine the level of diaphragm work during these experiments, we measured the TTI in animals ventilated using PSV and spontaneously breathing animals (Table 1). Note that TTI is often used to estimate both the mechanical load and energy use of the diaphragm during breathing [37]. By comparing the TTI between SB and PSV animals, we estimate that our trigger setting on the ventilator reduced the total work of spontaneous breathing by ~67%. That is, during PSV, the animals’ inspiratory muscles performed ~33% of the work of breathing.

### Comparison of Respiratory Work

**Table 1:** Comparison of respiratory work between spontaneously breathing animals (SB) and animals ventilated with PSV.

Parameter	SB group	PSV group
Ti (seconds)	0.175	0.050 *
Ttot (seconds)	0.877	1.048 *
Pneg (cm H_2_O)	-0.044	-0.058
TTI	0.0001082	0.00003599 *

Key: Ti = inspiratory time; Ttot = time of a breathing cycle; Pneg = inspiratory pressure; TTI—time tension index.

* indicates p<0.05.

### Systemic and biological response to MV

No significant differences existed in animal body weight between experimental groups before or after the experimental period. Heart rate (HR), systolic blood pressure (SBP), and body temperature (T) remained relatively constant during all MV protocols (HR range = 350–420 beats/min; SBP = 70–130 mmHg; T = 36–37°C). The arterial partial pressures of O_2_ (PaO_2_) and CO_2_ (PaCO_2_) were also reasonably constant during MV in all experimental groups. Specifically, PaO_2_ ranged from 65–100 mmHg whereas PaCO_2_ ranged from 32–42 mmHg. Importantly, at the completion of all MV protocols, there were no visual abnormalities of the lungs or peritoneal cavity, no sign of significant lung damage, and no evidence of infection.

### Mechanical ventilation-Induced oxidative stress

**Fig 1:**
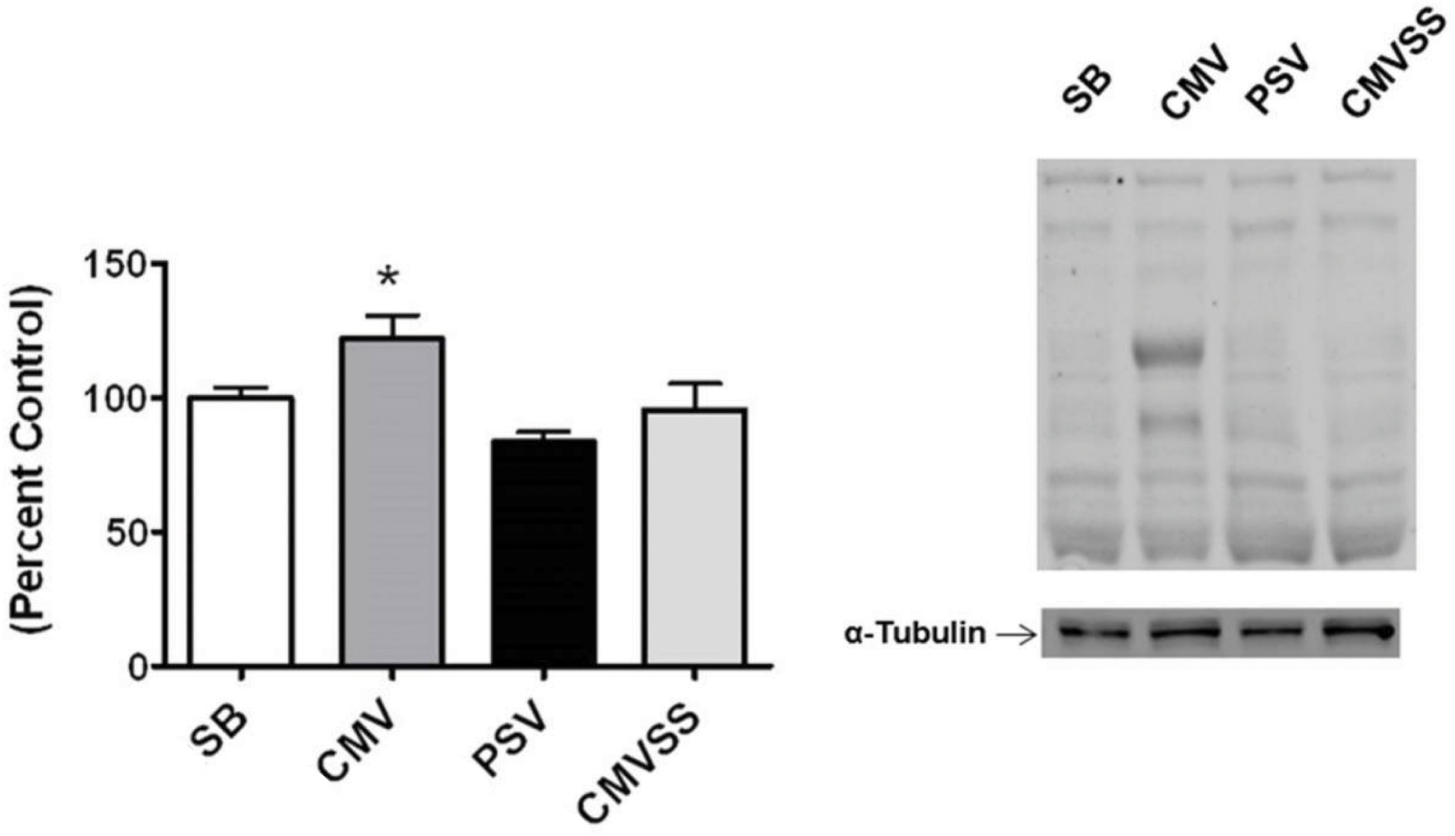
The levels of 4 hydroxynonenal (4-HNE) conjugated proteins in the diaphragm at 12 hours were measured via western blotting as an index of lipid peroxidation. A representative blot for 4-HNE protein conjugates is shown below the graph. The multiple bands represent different proteins modified by HNE-adducts, and give a general index of oxidative damage. Values are means ± SE and normalized to percent control. Symbols: * significantly different (p<0.05) from all other groups.

Lipid peroxidation occurs in cells in response to increased oxidant production and among the end products are several biologically active aldehydes (e.g., 4-HNE) that can be used as biomarkers of oxidative stress [38]. Our results indicate that 12 hours of CMV significantly increased diaphragmatic levels of 4-HNE (Fig 1). This is notable because oxidant stress is a required upstream trigger for the development of VIDD [9, 19, 24, 39–42]. Importantly, the current findings reveal that the use of PSV is sufficient to prevent ventilator-induced increases in 4-HNE in the diaphragm (p<0.05). Although 4-HNE is not a specific biomarker of mitochondrial generated ROS, the treatment of animals with the mitochondrial targeted antioxidant SS-31 is sufficient to prevent ventilator-induced increases in 4-HNE in the diaphragm (p<0.05) (Fig 1). This suggests that ventilator-induced increases in diaphragm lipid peroxidation occurred due to increased mitochondrial ROS production.

### Rates of diaphragm muscle protein synthesis

**Fig 2:**
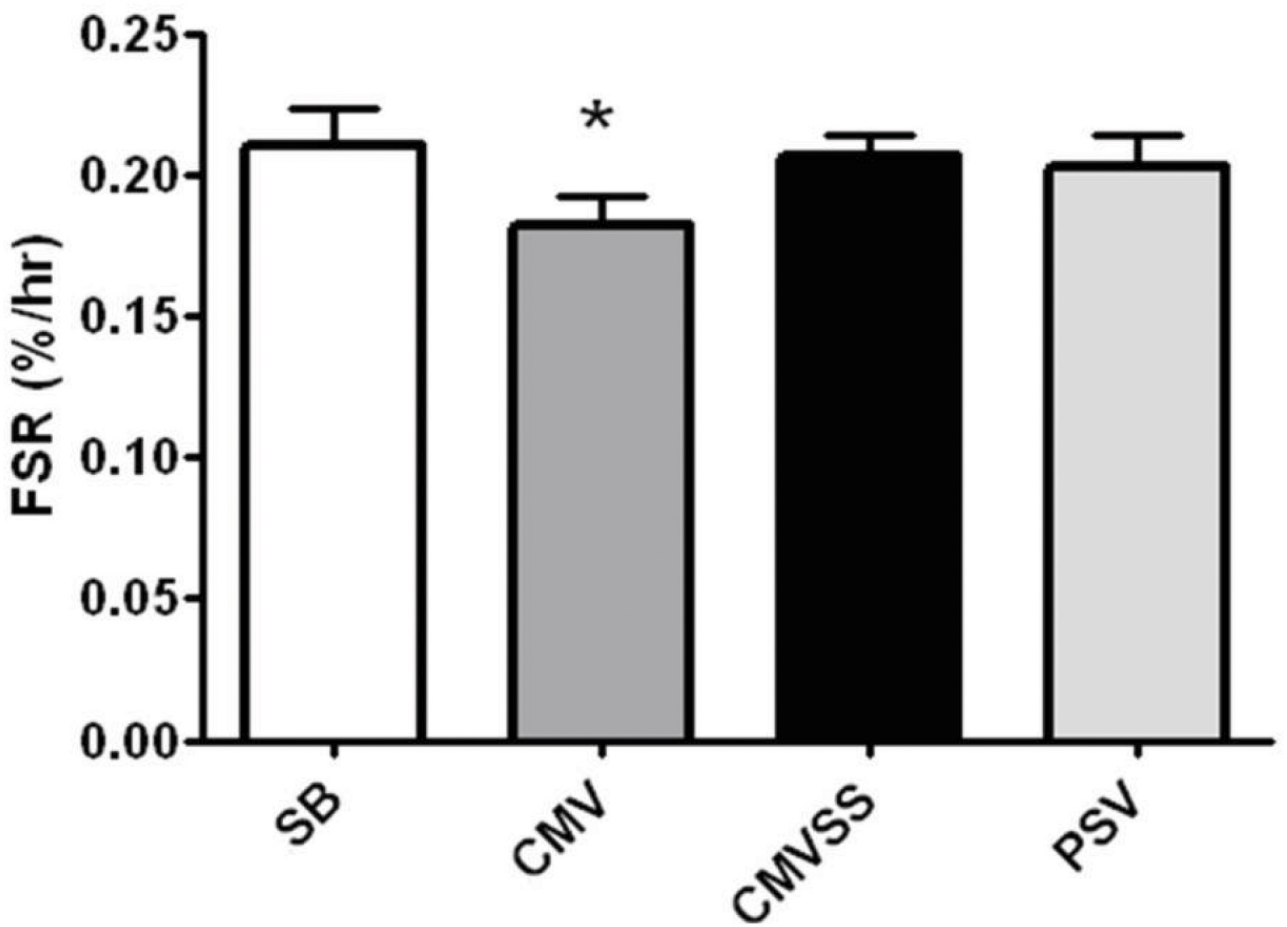
Mixed protein fractional synthesis rate (FSR) in the diaphragm following 12 hours of spontaneous breathing or mechanical ventilation. Values are expressed as percent change per hour (mean ± SE). Note that these 12 hour values represent an average FSR during 6–12 hours of the experiments. Symbols: * significantly different (p<0.05) from all other groups.

*In vivo* protein synthesis (i.e., fractional rate of mixed protein synthesis) in the diaphragm was measured by the incorporation of a labeled amino acid ([ring-^2^H_5_]-phenylalanine) into newly synthesized proteins. In agreement with previous work [8], our findings reveal that compared to anesthetized and time-matched spontaneously breathing animals, 12 hours of CMV results in a significant decrease in the rate of mixed muscle protein synthesis. Importantly, compared to CMV, PSV protected the diaphragm against CMV-induced decreases in diaphragm protein synthesis during ventilator support (Fig 2). Further, prevention of oxidative stress was successful in protecting against CMV-induced decreases in diaphragm protein synthesis during prolonged CMV (Fig 2).

### AKT/mTORC1 signaling in the diaphragm during prolonged MV

**Fig 3:**
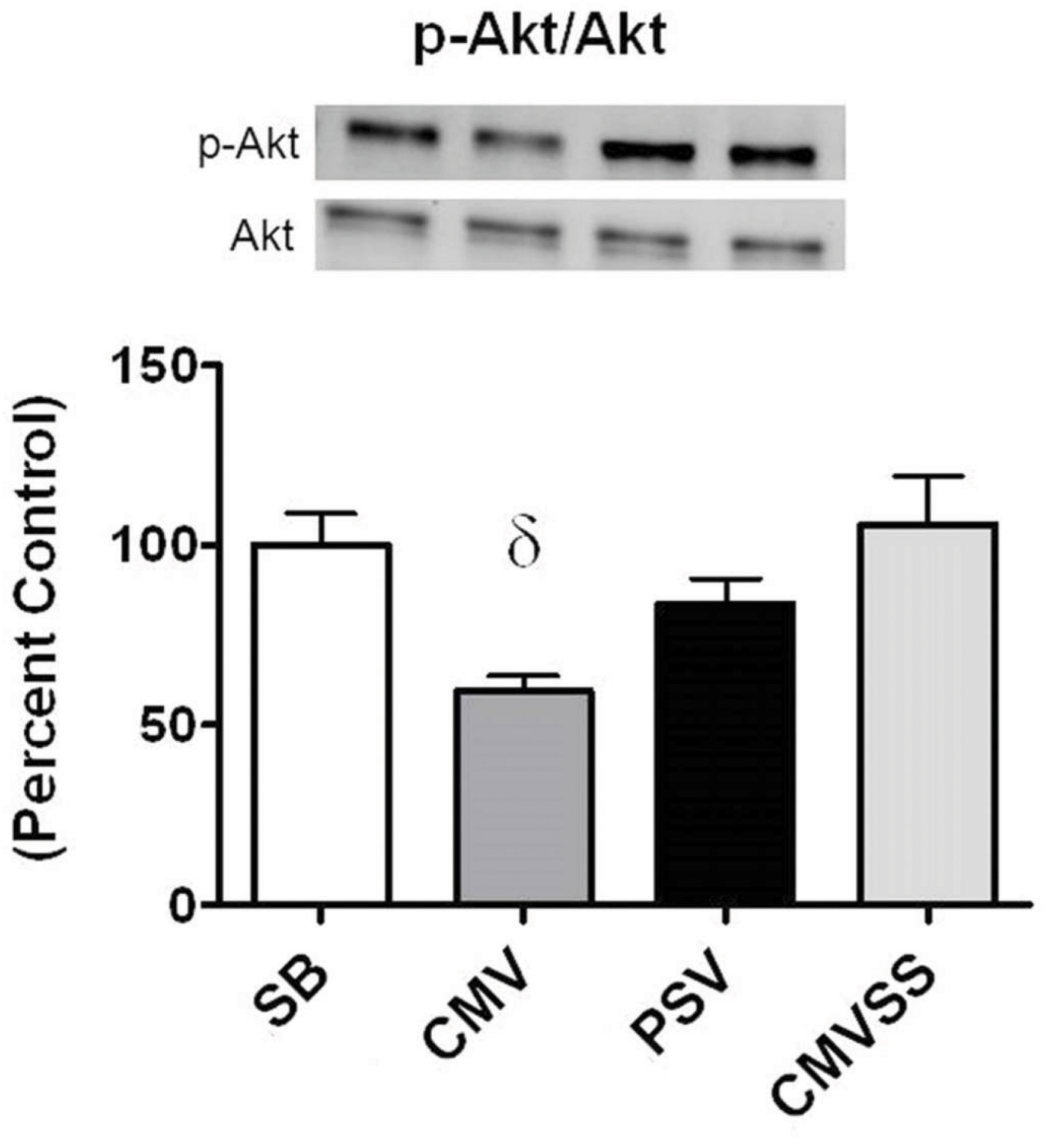
Ratio of p-AKT to total AKT protein in the diaphragm following 12 hours spontaneous breathing or mechanical ventilation. Representative western blots are shown below the graph. Values are expressed as (mean ± SE) change from percent control. Symbols: δ significantly different (p<0.05) from SB and CMVSS.

To gain insight into the effect of our experimental treatments on the signaling pathways that regulate diaphragm protein synthesis we measured key components of the AKT/mTORC1 signaling pathway. Specifically, active (phosphorylated) AKT (p-AKT) is a protein kinase that plays an important role in regulating muscle protein synthesis via regulation of mTORC1 and ultimately translation. Our results reveal that 12 hours of CMV significantly lowered the ratio of p-AKT to AKT in the diaphragm compared to SB, PSV, and CMVSS animals (p<0.05) (Fig 3). In contrast, no differences existed in the diaphragmatic p-AKT to AKT ratio between the SB, PSV, and CMVSS groups; this indicates that both interventions (i.e., PSV and mitochondrial antioxidant treatment) were successful in maintaining higher AKT phosphorylation in the diaphragm compared to the CMV animals. This finding is significant because active (i.e., phosphorylated) AKT has the potential to increase the rates of protein synthesis via activation of mTORC1. Indeed, activated mTORC1 plays a central role in promoting protein synthesis through the phosphorylation and activation of p70S6K and its target, rpS6, and phosphorylation of 4E-BP1 which removes the repression of eIF-4E on protein translation. Together, this cascade of events results in increased translation and thus increased rates of protein synthesis.

**Fig 4:**
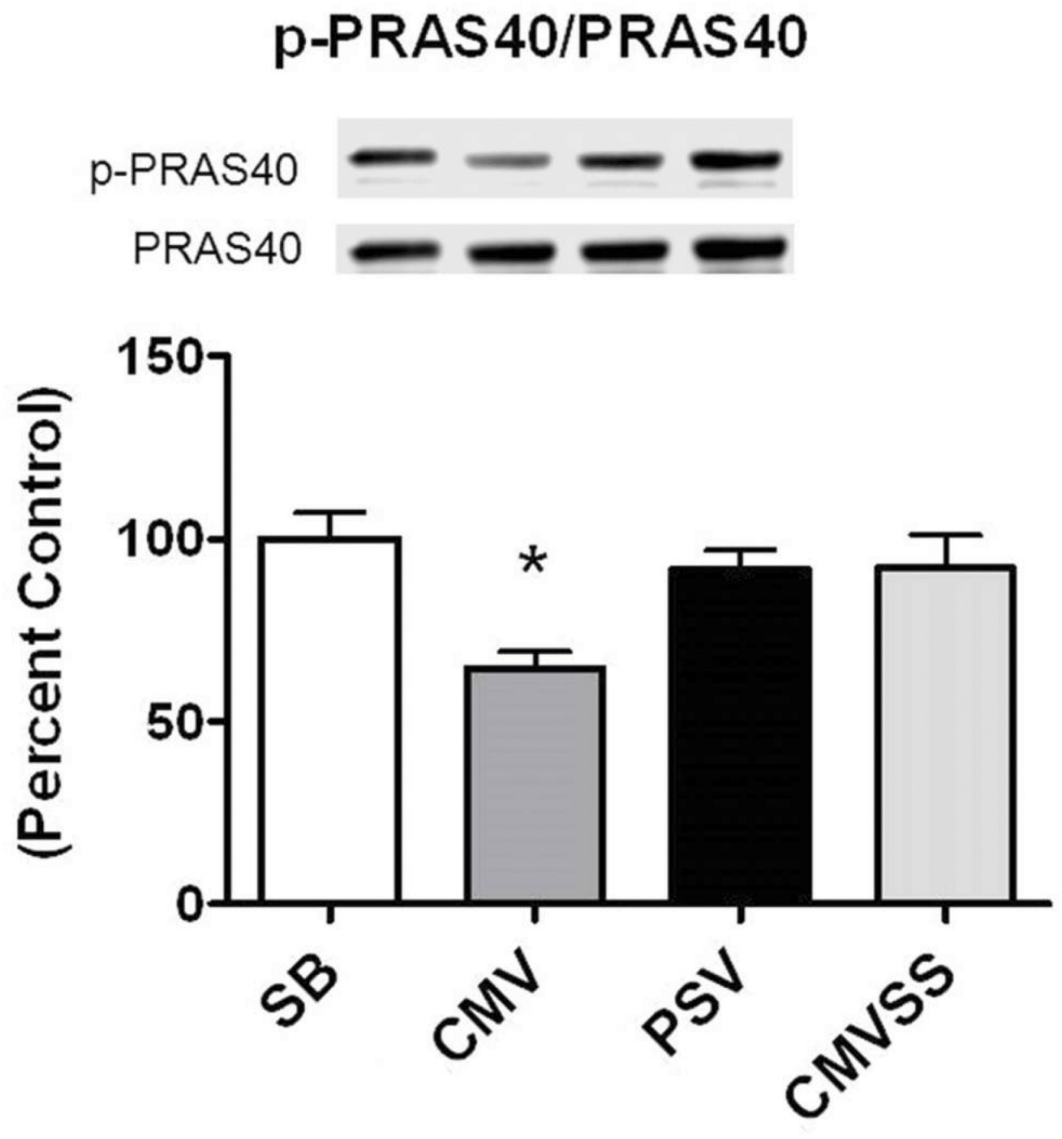
Ratio of p-PRAS40 to total PRAS40 protein after 12 hours spontaneous breathing or mechanical ventilation. Representative western blots are shown below the graph. Values are expressed as mean ± SE change from percent control. Symbols: * significantly different (p<0.05) from all other groups.

**Fig 5:**
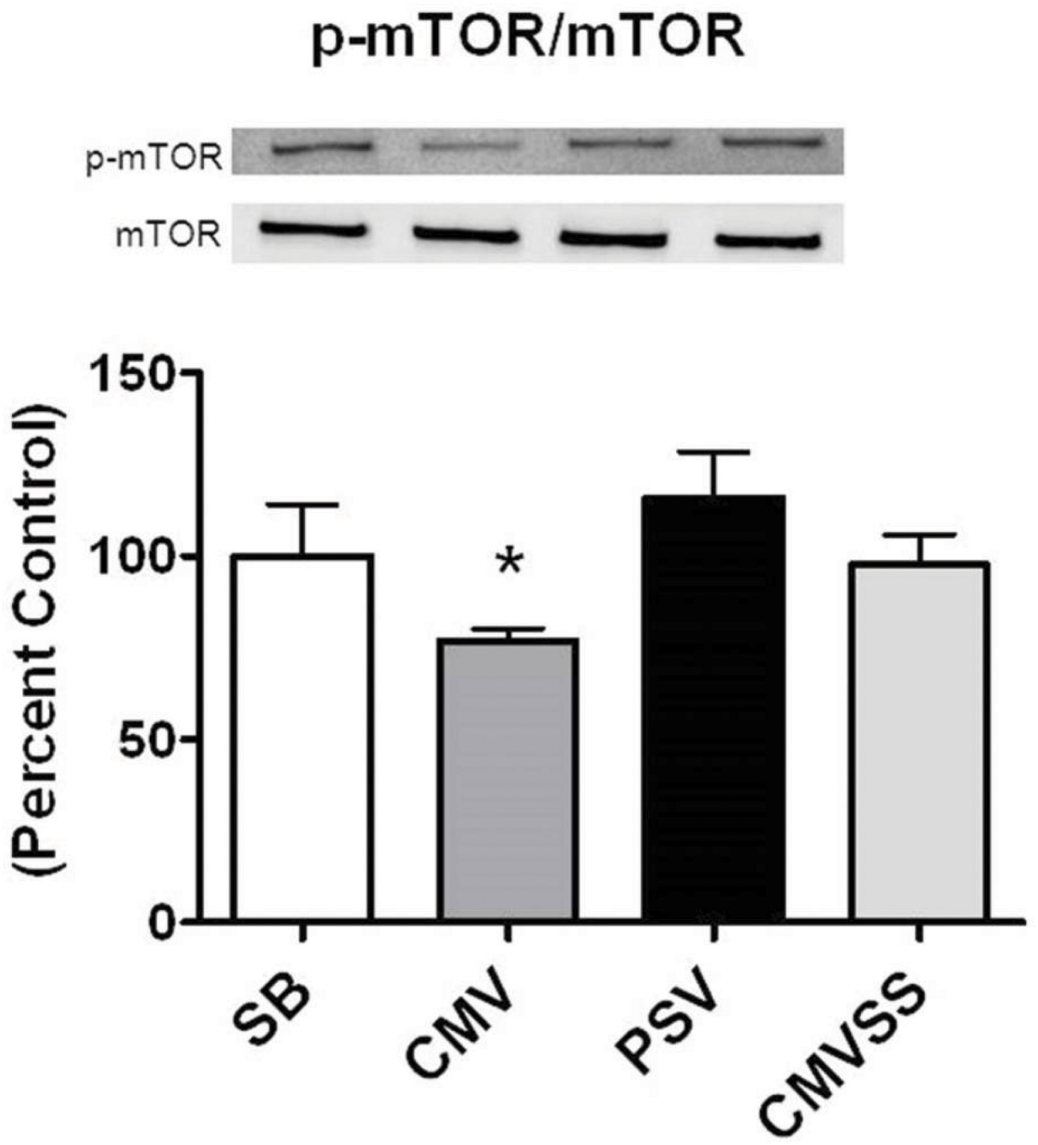
Ratio of p-mTOR to total mTOR protein in the diaphragm following 12 hours spontaneous breathing or mechanical ventilation. Representative western blots are shown below the graph. Values are expressed as mean ± SE change from percent control. Symbols: * significantly different (p<0.05) from all other groups.

In addition to direct phosphorylation of mTORC1, active AKT can also regulate mTORC1 activity through the phosphorylation of PRAS40 which facilitates mTORC1 activation by decreasing the binding affinity of PRAS40 to the mTORC1 complex [43]. In this regard, prolonged CMV decreased the ratio of both p-PRAS40/PRAS40 and p-mTOR/mTOR in the diaphragm (Figs 4 and 5). Nonetheless, it is unclear if these parallel changes in the phosphorylation status of PRAS40 and mTOR represent a cause and effect relationship.

**Fig 6:**
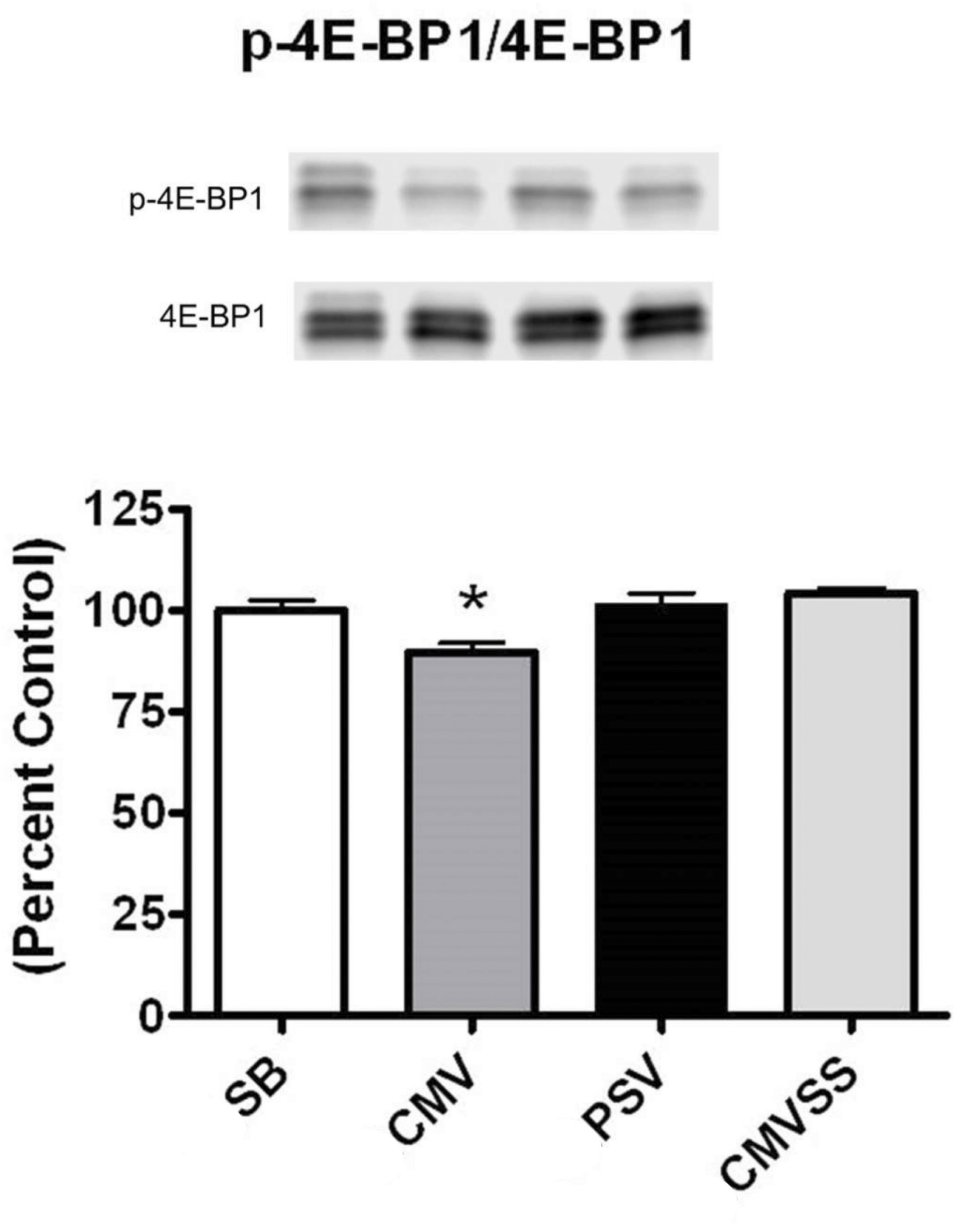
Ratio of p-4E-BP1 to total 4E-BP1 protein in the diaphragm following 12 hours of spontaneous breathing or mechanical ventilation. Representative western blots are shown below the graph. The ratio was determined via analysis of the sum total of all bands in both the phosphorylated and total blots. Values are expressed as mean ± SE change from percent control. Symbols: * significantly different (p<0.05) from all groups.

The protein 4E-BP1 is a substrate of mTORC1 and when activated by mTORC1, 4E-BP1 can promote the initiation of translation. Therefore, we measured the p-4E-BP1/4E-BP1 ratio in the diaphragm of all experimental groups. Our results reveal that compared to spontaneous breathing animals, prolonged CMV resulted in a significant reduction in the ratio of p-4E-BP1/4E-BP1 in the diaphragm (Fig 6). In contrast, the ratio of p-4E-BP1/4E-BP1 in the diaphragm did not differ between the spontaneous breathing animals and the PSV and CMVSS groups. Therefore, it is feasible that the higher ratio of p-4E-BP1/4E-BP1 contributed to the higher levels of protein synthesis in the diaphragms of the PSV and CMVSS animals.

## Discussion

### Overview of Principal Findings

Currently, there are no established therapeutic interventions to maintain protein synthesis in the diaphragm during prolonged ventilator support. The current experiments addressed this issue and provide novel findings regarding two divergent and beneficial approaches to maintain diaphragmatic protein synthesis during prolonged ventilator support. Specifically, our results reveal that compared to CMV, PSV protects against ventilator-induced decreases in the rate of diaphragm protein synthesis during the first 12 hours of ventilator support. Further, our data also show that prevention of MV-induced oxidative stress via treatment with a mitochondrial-targeted antioxidant guards against CMV-induced decreases in protein synthesis during 12 hours of CMV. A detailed discussion of these findings follows.

### PSV protects against the decreases in diaphragm protein synthesis that occurs during prolonged CMV

The current report is only the second investigation to compare the differences in diaphragm protein synthesis during both CMV and PSV. Futier et al. were the first investigators to report that PSV can preserve diaphragm protein synthesis during prolonged MV [22]. However, two experimental limitations exist in this report. First, *in vivo* diaphragm protein synthesis was not measured during prolonged mechanical ventilation, but instead diaphragm protein synthesis was assessed *in vitro* during a two-hour period following mechanical ventilation. Second, the level of ventilator support provided to the animals during prolonged mechanical ventilation was not determined and therefore the level of diaphragmatic contractile activity required to maintain diaphragm protein synthesis remains unknown. The current experiments were designed to eliminate these experimental drawbacks and provide new information missing from this previous report.

It is established that full support MV (i.e., CMV) results in dramatic diaphragm muscle atrophy in all fiber types in both humans and other animals [9, 44–48]. This CMV-induced diaphragmatic atrophy occurs due to increased proteolysis and decreased diaphragmatic protein synthesis [49]. Further, previous work reveals that compared to CMV, PSV can delay the rate of ventilator-induced diaphragm atrophy and contractile dysfunction [26]. The protective effects of PSV against ventilator-induced diaphragm atrophy are likely due to the fact that during PSV the diaphragm contracts and contributes to the work of breathing. In contrast, during CMV the diaphragm remains inactive as the ventilator provides all of the work of breathing. In the current experiments we assessed the work of breathing in both spontaneous breathing animals and animals supported by PSV. Our results indicate that compared to the work of spontaneous breathing, ventilating the animals using PSV reduced the work of breathing by ~67%. Nonetheless, this level of PSV was successful in protecting against the decrease in diaphragm protein synthesis that occurs during the first 12 hours of CMV.

To investigate the mechanism(s) responsible for the finding that PSV protects against CMV-induced decreases in diaphragm protein synthesis we measured a key biomarker of oxidative stress along with basic components of the AKT/mTOR signaling pathway. Compared to CMV, PSV averted the CMV-induced oxidative stress in the diaphragm following 12 hours of MV. This is important because oxidative stress can depress protein synthesis in several cell types [27–29]. Further, because the AKT/mTOR signaling pathway plays an important role in the regulation of cellular protein synthesis we measured diaphragmatic levels of key elements involved in this pathway. Specifically, the AKT/mTOR pathway and its downstream targets, ribosomal protein S6 and 4E-BP1, are immersed in the regulation of mRNA translation and activation of the AKT/mTOR pathway can increase protein synthesis in skeletal muscle fibers by promoting translation [50]. Compared to CMV, diaphragmatic levels of phosphorylated (active) AKT, phosphorylated (active) mTOR, and phosphorylated 4E-BP1 were higher in the PSV animals following 12 hours of MV. These findings are consistent with the observed higher rates of diaphragmatic protein synthesis during PSV compared to CMV. Collectively, these results indicate a general pattern of reduced anabolic signaling in the diaphragm following 12 hours of CMV that is averted using the level of PSV applied in these experiments. Interestingly, while the majority of patients in the ICU receive PSV many still develop significant diaphragm weakness. Since our results demonstrate that PSV maintains diaphragm protein synthesis, it is likely that the diaphragm weakness occurring in patients receiving PSV is due to protein degradation. This agrees with previous evidence that demonstrates that PSV can delay, but not prevent, increased protein degradation in the diaphragm [26].

### Mitochondrial-targeted antioxidant administration provides protection against MV-induced decreases in diaphragm protein synthesis

Oxidative damage to diaphragm lipids and proteins is a hallmark of VIDD (reviewed in [49]). Importantly, previous work indicates that prevention of MV-induced oxidative stress via antioxidants can defend against MV-induced diaphragm atrophy and contractile dysfunction [9, 24, 39, 41]. More specifically, recent studies reveal that increased mitochondria ROS production during MV is a required upstream signal to promote diaphragm dysfunction in both animals and humans [21, 51]. Further, inhibition of MV-induced mitochondrial ROS emission via administration of the mitochondrial-targeted antioxidant SS-31 can protect against both diaphragm atrophy and contractile dysfunction in rats [19]. This previous study reveals that SS-31 protects the diaphragm against increased proteolysis by inhibiting the activation of several proteolytic systems (i.e. calpain, caspase-3 and the ubiquitin-proteasome system) [19]. Moreover, it is feasible that prevention of oxidative stress in the diaphragm can preserve the rate of *in vivo* protein synthesis in the diaphragm during CMV. This prediction is based on evidence indicating that increased oxidative stress is sufficient to depress the rates of protein synthesis [27–29]. The current results reveal that treatment of animals with SS-31 successfully prevented MV-induced oxidative stress in the diaphragm and protected against the MV-induced decrease in diaphragm protein synthesis during 12 hours of CMV.

To investigate potential mechanisms responsible for this finding, we determined the impact of the mitochondrial-targeted antioxidant SS-31 on AKT/mTORC1 signaling in the diaphragm during prolonged CMV. Treatment with the mitochondrial targeted antioxidant prevented the MV-induced decline in the activity of AKT, mTOR, and 4E-BP1 in the diaphragm. These data suggest that treatment of animals with a mitochondrial-targeted antioxidant is not only able to prevent the activation of key proteolytic enzymes in the diaphragm, but is also sufficient to maintain protein synthesis possibly by maintaining activation of the AKT/mTORC1 signaling pathway in the diaphragm.

## Conclusions

These experiments attempted to identify therapeutic interventions that are capable of supporting protein synthesis in the diaphragm during prolonged CMV. Importantly, our results reveal that compared to CMV, partial support mechanical ventilation (i.e., PSV) can protect against ventilator-induced decreases in the rate of diaphragm protein synthesis during the first 12 hours of ventilator support. Further, our data also demonstrate that treatment with a mitochondrial-targeted antioxidant guards against CMV-induced decreases in protein synthesis during 12 hours of CMV. The mechanism(s) responsible for the PSV and mitochondrial antioxidant-mediated protection against depressed diaphragm protein synthesis are not completely clear. Nonetheless, both experimental interventions (i.e., PSV and mitochondrial-targeted antioxidant) prevent MV-induced oxidative stress in the diaphragm and results in higher activity of Akt, mTOR, and 4E-BP1 in the diaphragm during 12 hours of prolonged MV. Additional work is required to determine if these mechanisms are solely responsible for the maintenance of protein synthesis in the diaphragm, and also to determine the effects of combining both interventions. Further, future studies are needed to determine what specific proteins, such as contractile proteins, are preserved through maintaining protein synthesis. Regardless of mechanisms or proteins are involved our findings suggest that the use of PSV and/or mitochondrial-targeted antioxidants has the potential to maintain diaphragmatic protein synthesis during prolonged ventilator support. This is important because maintaining protein synthesis in the diaphragm during prolonged MV can protect against ventilator-induced diaphragmatic atrophy and weakness.
